# Recognition and management of adverse cutaneous reactions in patients on enfortumab vedotin and pembrolizumab in the inpatient setting

**DOI:** 10.1016/j.jdcr.2024.12.037

**Published:** 2025-01-28

**Authors:** Emma L. Myers, Jessica Liu, Sabrina M. Shearer, Sarah A. Myers, Michelle Schneider, Christopher Hoimes, Melodi Javid Whitley

**Affiliations:** a University of North Carolina School of Medicine, Chapel Hill, North Carolina; b Duke University School of Medicine, Durham, North Carolina; c Department of Dermatology, Duke University School of Medicine, Durham, North Carolina; d Department of Pathology, Duke University School of Medicine, Durham, North Carolina; e Division on Medical Oncology and Center for Cancer Immunotherapy, Department of Medicine, Duke University School of Medicine, Durham, North Carolina

**Keywords:** enfortumab, immune checkpoint inhibitor, immunotherapy, oncodermatology, pembrolizumab, severe cutaneous adverse reaction

## Introduction

Metastatic urothelial carcinoma (mUC) is an aggressive malignancy with historically poor survival rates with platinum-based chemotherapy. Recently, combination therapy with nectin-4-directed antibody-drug conjugate enfortumab vedotin (EV) and programmed death-1 (PD-1) inhibitor pembrolizumab (P) has emerged as a preferred first-line therapy, demonstrating superior progression-free and overall survival.^1^^,^^2^ However, both EV and P have been associated with frequent cutaneous adverse events (cAEs), including widespread toxic erythema of chemotherapy (TEC), Stevens-Johnson syndrome (SJS), and toxic epidermal necrolysis (TEN).^3^, ^4^, ^5^, ^6^, ^7^, ^8^, ^9^, ^10^ Here, we present 8 cases demonstrating the clinical and histologic spectrum and management of cAEs in patients on EV + P therapy that present in the inpatient setting.

## Methodology

We conducted an institutional review board approved single-institution retrospective chart review to identify hospitalized patients receiving EV + P therapy who were evaluated by the inpatient dermatology consultation service. Medical records were reviewed to assess patient characteristics, presentation, clinical and histologic features, laboratory parameters, and outcomes.

## Results

### Baseline characteristics

This case series consisted of an equal number of females (*n* = 4) and males (*n* = 4) with an average age of 72.3 years (mean (M) = 72.3, range (R) = 63,86) (Table I). The cohort included Asian (*n* = 1), Black (*n* = 3), and White (*n* = 4) individuals, all of whom identified as non-Hispanic/Latino (*n* = 8). All patients were diagnosed with metastatic urothelial cancer except for P_3_ and P_6_ with stage II (P_6_) and III (P_3_) locally advanced/unresectable urothelial cancer, indicating bladder muscle invasive disease only. Four patients had prior exposure to different chemotherapeutic or immunomodulatory treatments and the rest received EV + P as first-line therapy. One patient, P_3,_ had a history of cutaneous drug rash from allopurinol; all others (*n* = 7) had no prior history.

**Table I:** Demographics of patients that experienced cAEs while receiving EV + P

Patient	P_1_	P_2_	P_3_	P_4_	P_5_	P_6_	P_7_	P_8_
Age	76	69	63	66	86	86	67	65
Sex	M	M	M	F	F	F	M	F
Race	White	White	White	Black	White	Asian	Black	Black
Oncologic Hx								
Cancer type	mUC	mUC	laUC	mUC∗	mUC	laUC	mUC∗	mUC
Tumor location	Bladder	Upper tract	Bladder	Bladder	Ureter	Bladder	Bladder	Bladder
Stage at time of. EV + P initiation†	IV	IV	III	IV	IV	II	IV	IV
Treatments prior to EV + P	- Mito - BCG + Gem - NA GemCis‡ + RC	- GemCis - RT§	None	MVAC	None‖	None	None	- GemCis - Avelumab
Previous hx of drug reaction?	-	-	+¶	-	-	-	-	-

*BCG*, Bacillus Calmette-Guerin; *Gem*, gemcitabine; *GemCis*, gemcitabine plus cisplastin; *Hx*, history; *Mito*, mitomyocin; *MVAC*, methotrexate, vinblastine sulfate, doxorubicin hydrochloride (Adriamycin), and cisplastin; *NA*, neoadjuvant therapy; *RC*, radical cystectomy.

∗ Plasmacytoid type.

† Tumor staging according to National Comprehensive Cancer Network bladder cancer staging guidelines.

‡ NA to radical cystectomy.

§ For bone metastasis.

‖ s/p left distal ureterectomy.

¶ Allopurinol; seborrheic dermatitis & erythroderma.

### Presentations

All patients experienced a milder cutaneous eruption preceding hospitalization characterized by generalized pruritus without rash or focal pruritic maculopapular eruptions, with initial symptoms occurring after their first cycle of EV + P. Three patients developed symptoms after C1D1 and 5 patients developed symptoms after C1D8. Most patients presented to the hospital with progressive dermatologic symptoms early in their treatment course, following Cycle 1 (*n* = 4) or Cycle 2 (*n* = 3). Patients presented to the hospital 9 days (M = 9.4, R = 1,21) after their last dose of EV + P and 29 days after the milder cutaneous eruption (M = 29, R = 3106). Dermatology was promptly consulted (*n* = 5 consulted within 1 day of admission; M = 1.8 days after admission, R = 0, 8 days). At the time of the inpatient dermatology consult, most patients demonstrated signs of systemic illness or organ dysfunction, including acute kidney injury (*n* = 2), transaminitis (*n* = 4), fever (*n* = 2), or infection (*n* = 1 with urosepsis, *n* = 1 with bacteremia) in addition to the principal cutaneous findings. No patients demonstrated peripheral eosinophilia (*n* = 0) (Table II).

**Table II:** Clinical and pathologic presentations at time of inpatient dermatology consult

Patient	P_1_	P_2_	P_3_	P_4_	P_5_	P_6_	P_7_	P_8_
CTx cycle at symptom onset∗†	C1D1	C1D8‡	C1D8	C1D8	C1D1	C1D8	C1D8	C1D1
CTx cycle on admission	C1D8	C6D8	C1D8	C2D8	C2D8	C1D8	C2D8	C1D8
Days from last dose to admission	1	6	10	12	6	9	21	10
Days from admission to dermatology consult	8	1	0	1	0	1§	1	0‖
Systemic involvement¶								
Fever	+	-	-	-	-	-	+	-
Leukocytosis	+	-	-	-	-	-	+	-
Infection	+	-	-	-	+	-	-	-
Tachycardia	-	-	+	-	-	-	+	+
Hypotension	-	-	+	-	-	-	-	+
AKI	-	-	+	-	-	-	+	+
Transaminitis	-	-	+	-	+	+	+	-
Elevated ESR/CRP	-	-	+	-	+	-	-	-
Elevated lactate	-	+	-	-	-	-	+	-
Rash gross morphology¶								
Morbilliform eruption	+	+	+	+	+	+	+	+
Diffuse erythroderma	-	-	-	-	-	-	+	+
Bullae	-	-	-	-	+	+	+	+
Positive Nikolsky sign	-	-	-	-	-	+	+	+
Rash distribution¶								
Head/neck	-	-	-	-	-	-	+	+
Trunk	+	+	+	+	+	+	+	+
Extremities	+	+	+	+	+	+	+	+
Acral	-	-	-	-	+	-	+	-
Intertriginous	-	-	-	+	+	+	+	+
Genital	-	-	-	+	-	-	+	+
Mucosal (oral)	-	-	+	-	-	+	+	+
Mucosal (ocular)	-	+	-	-	-	-	+	-
Brief pathology report								
Spongiosis	*N/A*	+	+	*N/A*	*N/A*	+	-	-
Perivascular infiltration		+	+			**+**	-	**+**
Eosinophils		-	**+**			**+**	**-**	
Interface dermatitis		-	-			**+**	**+**	**+**
Full thickness necrosis		-	-			**+**	**+**	**+**
Partial thickness necrosis		-	+			-	-	-
Eccrine involvement		-	-			**+**	**+**	-
DIF	*N/A*	*N/A*	*N/A*	*N/A*	*N/A*	*N/A*	negative	negative
Severity¶								
Grade#	3	3	3	2	3	4	4	4
SCORETEN	*N/A*	*N/A*	*N/A*	*N/A*	3	2	6	4

*AKI*, Acute kidney injury; *BSA*, body surface area; *CRP*, C-reactive protein; *CTx*, chemotherapy; *DIF*, direct immunofluorescence; *ESR*, erythrocyte sedimentation rate; *N/A*, not available.

∗ Mild rashes are documented in 48% of trial patients receiving EV, typically occurring 15.9 days after initiation.

† A typical course of EV + P includes 12 cycles, as reported in the phase III EV-301 Trial (NCT03474107)1. One cycle of EV + P is 21 days, with EV dosed on D1 and D8 and P dosed only on D1.

‡ EV only on C1D1 pending insurance approval of pembrolizumab.

§ Excluding 3 day stay at outside hospital awaiting transfer for inpatient dermatology evaluation.

‖ Excluding 2 day stay at outside hospital awaiting transfer for inpatient dermatology evaluation.

¶ Presentation described on day of inpatient dermatology consult.

# Definition of grades of severity for various common irAEs. These definitions have been taken from Common Terminology Criteria for Adverse Events, Version 5.0.

**Fig 1:**
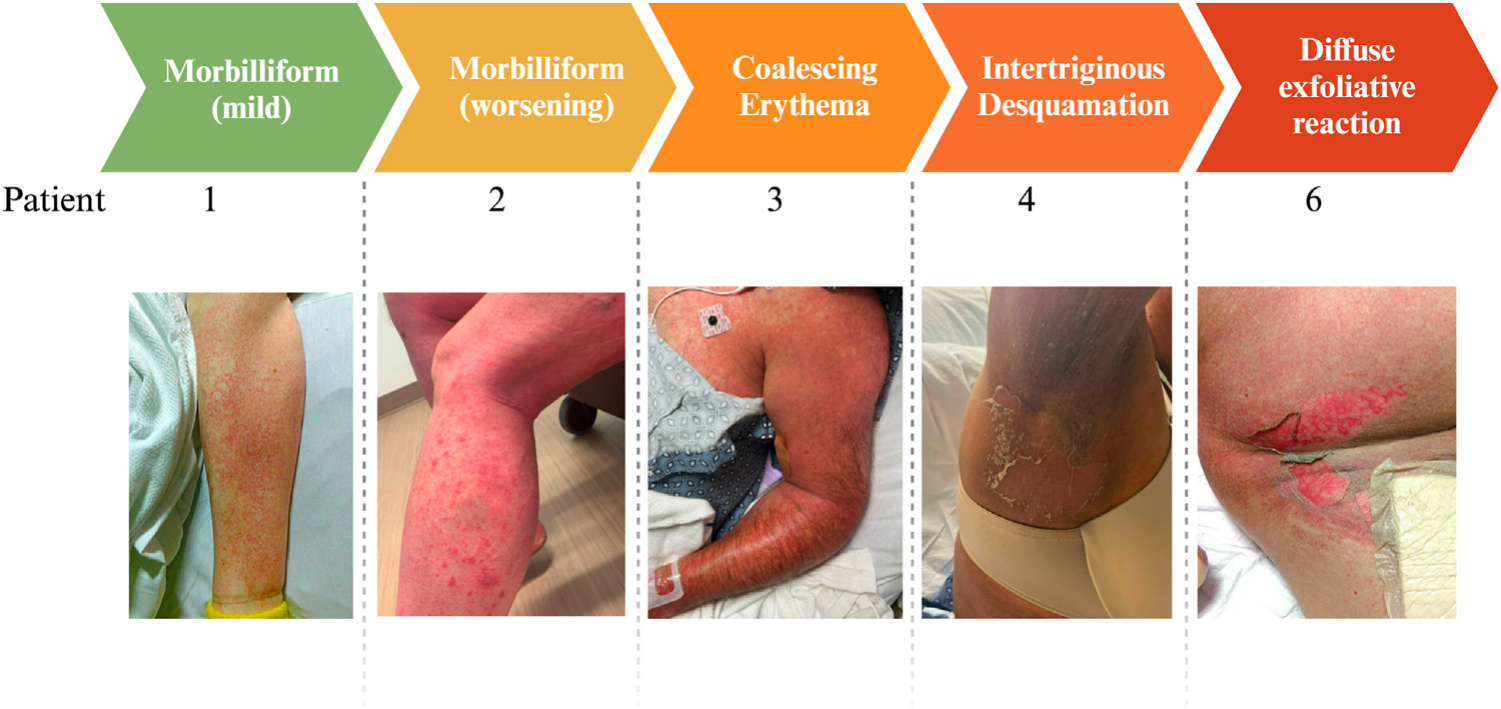
Spectrum of clinical images.

All 8 patients presented with morbilliform eruption (Table II, Fig 1). Milder eruptions were consistent with exanthematous drug eruptions (P_1-5_), while more severe reactions were in the clinical spectrum of TEC, SJS, or TEN (P_6-8_). P_6-8_ had notably severe presentations (Grade 4), including cutaneous findings of serous/hemorrhagic bullae (*n* = 3), positive Nikolsky sign (*n* = 3), and diffuse erythroderma (*n* = 1) (Fig 1). Most experienced involvement of the extremities (*n* = 8), trunk (*n* = 8) and intertriginous sites (*n* = 5), with few patients experiencing head/neck (*n* = 2), genital (*n* = 3), acral (*n* = 2), and mucosal involvement (*n* = 5; oral *n* = 4; ocular *n* = 2). Mucosal involvement was noted in all Grade 4 cAEs (P_6-8_) and in 2 patients with milder disease (P_2,3_) (Table II).

**Fig 2:**
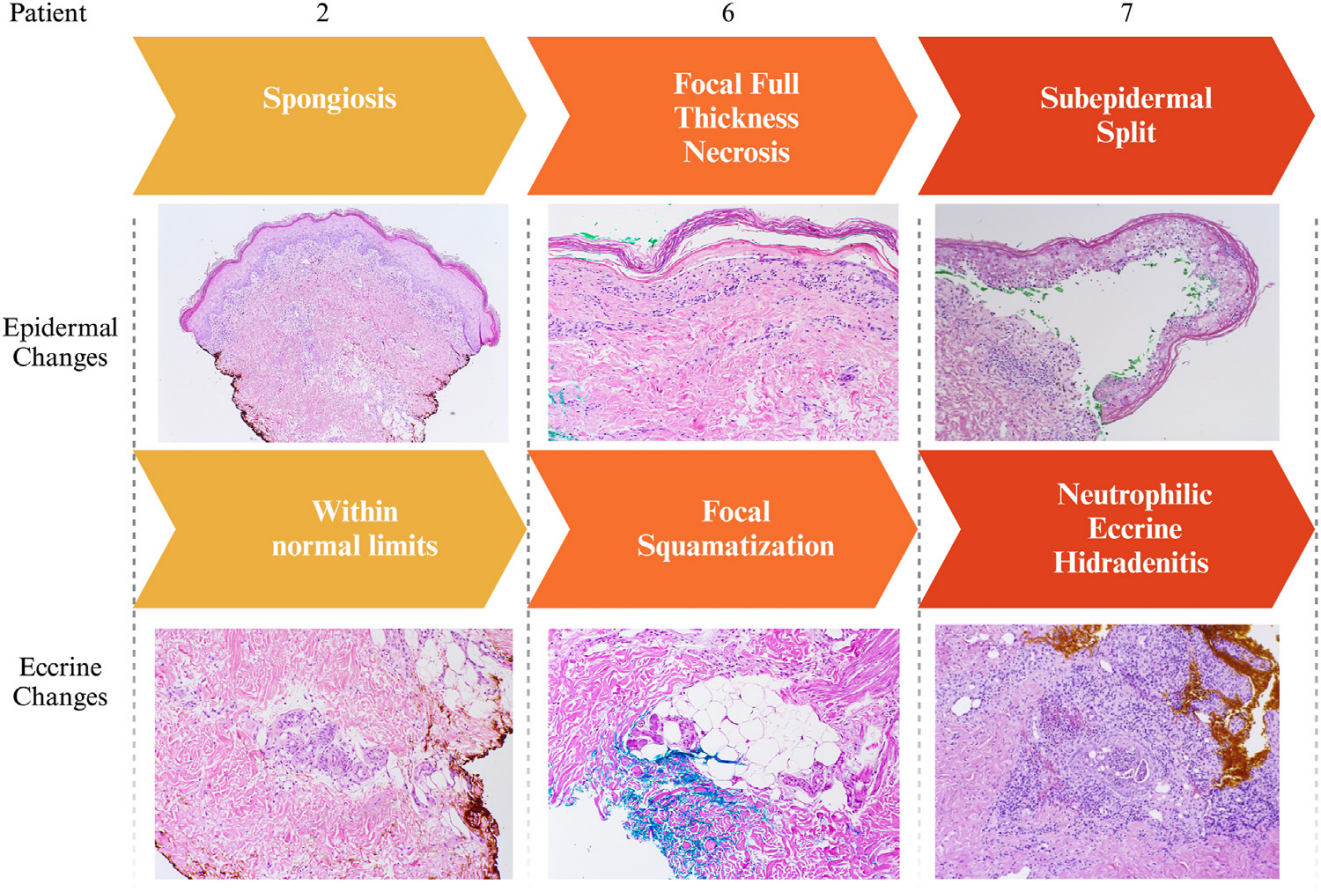
Spectrum of pathology findings.

Most patients underwent skin biopsies (*n* = 5). On histopathologic examination, P_6-8_ demonstrated interface dermatitis and focal full thickness epidermal necrosis (*n* = 3 of 3) with one patient sample demonstrating eosinophils (*n* = 1 of 3) and 2 demonstrating eccrine involvement (*n* = 2 of 3) (Fig 2). Patients with milder disease (Grade 2 or 3) exhibited spongiotic dermatitis, with one case demonstrating eosinophils. Additional findings were perivascular infiltration (*n* = 4) and partial thickness necrosis (*n* = 1).

### Treatments and outcomes

Prior to admission, all patients trialed corticosteroids (*n* = 6 topical, *n* = 2 oral, *n* = 2 intravenous (IV)/intramuscular). During admission, most received systemic corticosteroids (*n* = 2 oral only, *n* = 4 both oral and IV, *n* = 1 patient expired prior to receiving IV), initiated within 24 hours of admission. Patients with Grade 4 reactions (*n* = 3) received additional systemic therapies, including intravenous immunoglobulin (*n* = 2), rifampin (*n* = 1), and antibiotics (*n* = 3). One patient was transferred to a burn unit for specialized care (*n* = 1). Patients were admitted for an average of 8 days (M = 7.8, R = 1, 28). Most patients demonstrated clinical improvement in skin symptoms prior to discharge (*n* = 5 improved before discharge, *n* = 1 improved after discharge, *n* = 1 not documented, *n* = 1 died). Of patients who demonstrated clinical improvement, the first documented improvement occurred 5 days after admission (*n* = 6, M = 5.4, R = 2, 10) (Table III). Almost all patients achieved complete resolution of their cutaneous reaction (*n* = 6, *n* = 1 not documented). One patient expired due to multiorgan failure within 24 hours of hospitalization. Surviving patients achieved complete resolution around 29 days (*n* = 6, M = 28.8, R = 9, 50). At the time of complete resolution, almost all surviving patients were on oral prednisone (*n* = 6). Patients completed their steroid regimen 25 days after discharge (*n* = 4, M = 25, R = 15,43). At time of submission, 2 patients had yet to complete their prednisone regimen (*n* = 2).

**Table III:** cAE treatments and clinical/oncologic outcomes

Patient	P_1_	P_2_	P_3_	P_4_	P_5_	P_6_	P_7_	P_8_
Treatments prior to admission								
Topical steroids	**+**	**+**	**+**	**+**	**+**	**+**		
Antihistamines		**+**		**+**	**+**∗		**+**	**+**
Systemic steroids				**+**			**+**	**+**
Antibiotics			**+**†					
Systemic therapies inpatient								
Antihistamines	**+**		**+**	**+**	**+**∗	**+**	**+**	
Oral steroids		**+**	**+**	**+**	**+**‡	**+**	**+**	
IV steroids			**+**	**+**		**+**	**+**	§
IVIG‖						**+**		**+**
Rifampin							**+**	§
Cyclosporine								**+**
Antibiotics	**+**¶				**+**#	**+**	**+**	**+**
Burn unit care							**+**	§
Max steroid dose (prednisone equivalent, mg/kg)	N/A	0.7	1.0	1.3	0.6	2.4	2.9	N/A
Duration of admission	16	5	4	3	11	10∗∗	28††	1‡‡
Days to first improvement§§	N/A‖‖	2	2	9	4	3∗∗	10	N/A
Days to complete resolution¶¶	N/A‖‖	50	18	9	36	16	44	N/A
Duration of steroid taper##	N/A	Ongoing∗∗∗	26	15	Ongoing†††	16	43	N/A
Rechallenged with EV + P	**+**	**+**	**+**	**+**	-	-	-	N/A
Days to EV + P rechallenge‡‡‡	5	25	29	7	N/A	N/A	N/A	N/A
EV dose reduction	-	**+**	-	**+**	N/A	N/A	N/A	N/A
Subsequent flares with EV + P	**+**	**+**	-	**+**	N/A	N/A	N/A	N/A
Subsequent cancer treatment	EV + P, TARE	EV + P, TARE	EV + P, RC	EV + P	P§§§	None	Avelumab∗∗∗∗	N/A
Duration of follow-up‖‖‖	175	471	454	309	274	72	258	N/A
Current cancer status	PR	PR	CR	CR	CR	CR¶¶¶	CR###	PD

*CR*, Complete remission; *NR*, no response; *PD*, patient death; *PR*, partial response; *TAC*, triamcinolone; *TARE*, transarterial radioembolization (for liver metastases).

∗ Home dose.

† For sore throat, unclear etiology of pharyngitis v. mucositis.

‡ Increased from baseline low dose prednisone for COPD.

§ Patient expired before treatment initiation.

‖ P_6_ received 2 doses (20g each) of 0.4 g/kg IVIG over 2 days; IV steroids were continued throughout doses of IVIG. P_8_ received a single dose (66.2 g) of 1 g/kg IVIG and expired on day of transfer to our institution, prior to intended initiation of systemic steroids.

¶ For concurrent bacteremia secondary to UTI.

# For positive blood cultures on admission with possible port site etiology, discontinued after negative surveillance cultures.

∗∗ Excluding 3 day stay at outside hospital awaiting transfer for inpatient dermatology evaluation.

†† Including 12 day interval stay at burn center.

‡‡ Excluding 2 day stay at outside hospital awaiting transfer for inpatient dermatology evaluation.

§§ Days from dermatology consult to date of first documented clinical improvement.

‖‖ Not documented.

¶¶ Days from dermatology consult to date of documented complete or near complete resolution of cutaneous symptoms.

## Days from discharge to end of steroid taper.

∗∗∗ On long term low dose prednisone after multiple failed attempts to taper due to rash flares.

††† Increased from baseline for rash and COPD flares throughout course of pembrolizumab therapy, ongoing titration for COPD.

‡‡‡ Days from discharge to first day of subsequent EV + P therapy.

§§§ No cutaneous flares on pembrolizumab monotherapy, initiated 26 days after discharge.

‖‖‖ Days from dermatology consult to last known dermatology or oncology follow-up.

¶¶¶ Imaging without evidence of recurrence or metastasis, pending staging by oncologist.

### With concern for recurrence.

∗∗∗∗ No cutaneous flares on avelumab maintenance therapy, initiated 134 days after discharge.

All surviving patients suspended EV + P treatment due to cutaneous toxicity (*n* = 7). Following stabilization, most were rechallenged (*n* = 4 resumed EV + P, *n* = 1 switched to P monotherapy). One patient was not rechallenged due to suspected oncologic remission. No Grade 4 cases were rechallenged with EV + P; though one (P_7_) started avelumab (PD-1 inhibitor) for maintenance therapy, and tolerated it well without rash recrudescence. Of patients who resumed EV + P (*n* = 4), 3 experienced cutaneous flares with subsequent doses of EV + P, and 2 resumed at reduced EV doses to reduce the risk of skin toxicity. One patient discontinued EV and resumed with P monotherapy due to skin toxicity. All cutaneous flares were managed successfully in the outpatient setting (*n* = 3), with one patient on chronic low-dose steroids to manage recurrent skin symptoms. Most patients have demonstrated clinical improvement or complete remission of their urothelial cancer (*n* = 2 partial response, *n* = 5 complete remission, *n* = 1 death) (Table III).

## Discussion

**Fig 3:**
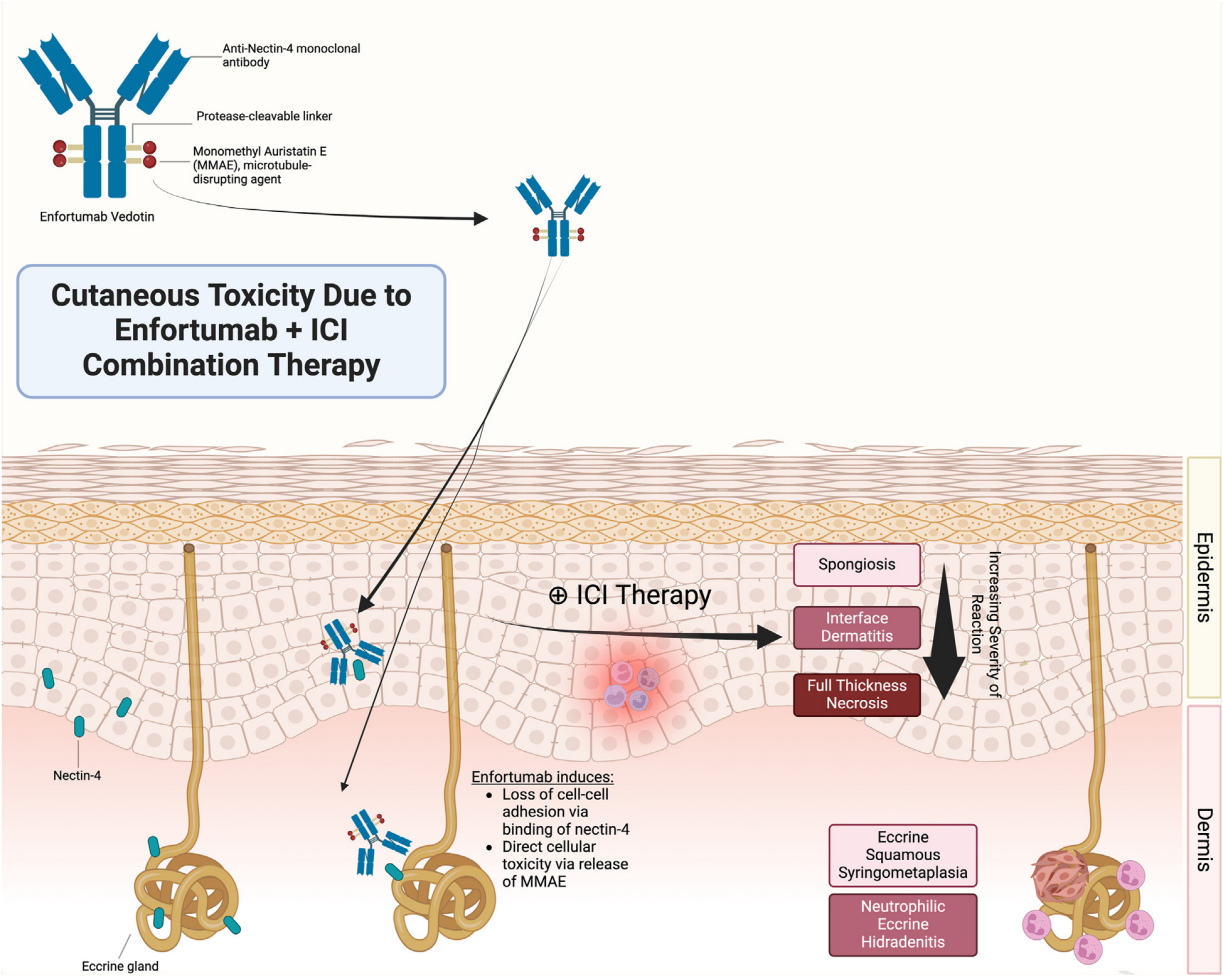
Cutaneous toxicity due to enfortumab + ICI combination therapy.

Both EV and P commonly trigger cAEs via distinct mechanisms. EV is an antibody-drug conjugate composed of cytotoxic microtubule inhibitor, monomethyl auristatin E (MMAE), and a monoclonal antibody targeting nectin-4 (Fig 3). Nectin-4 is a cell adhesion molecule expressed in various epithelial malignancies including urothelial carcinoma, as well as in normal skin tissues, specifically in basal keratinocytes, sweat glands, and hair follicles, thus predisposing patients to cutaneous reactions.^7^ Actively dividing epidermal keratinocytes may be particularly susceptible to the antimitotic effects of MMAE, suggesting a combined effect of nectin-4-induced keratinocyte disruption and MMAE-induced toxicity (Fig 3). In phase II and III clinical trials, 55% of patients on EV monotherapy for urothelial cancer developed treatment-related skin reactions, and 13% of patients developed Grade 3 or higher reactions.^7^ Dermatologic reactions range from maculopapular eruptions to severe adverse reactions such as TEC and SJS/TEN, with a median time of onset of 0.427 months (0.03-12.68) as reported in the phase III EV-301 Trial (NCT03474107).^1^

PD-1 inhibitors such as P are ICIs that produce antitumor effects by removing inhibitory signaling on the immune system, thus promoting T-cell responses against tumor cells. This immune activation also frequently results in autoinflammatory effects on noncancerous tissues, including cAEs.^9^ Over one third of patients treated with PD-1 inhibitors develop cAEs, most commonly eczematous, maculopapular, and lichenoid dermatoses. Although ICI-induced SJS/TEN cases are rare, reactions can be life-threatening with a highly variable time to onset, ranging from weeks to months after ICI initiation.^9^ Importantly, patients on combination therapy with EV + P are diagnosed on a clinical spectrum that encompasses overlapping features of SJS/TEN and TEC, as it is difficult to differentiate the primary process driving the cutaneous features. Future translational studies that compare the transcriptomic and proteomic signatures of patients with known TEC versus SJS/TEN may provide more definitive evidence of the primary disease process.

Combination therapy with EV + P appears to result in higher incidence of treatment-related cutaneous adverse events. In the pivotal EV + P trial, 67.1% of patients randomized to receive combination therapy developed any grade skin reactions, and 21.1% developed Grade 3 or higher reactions, compared to 45.2% and 8.2% in the EV monotherapy arm.^11^ The reason for the increase in frequency and severity of cutaneous reactions on combination therapy is unclear; an ICI-priming effect has been proposed.^4^

Our patient case series demonstrates the spectrum of moderate to severe cutaneous adverse reactions that may occur in patients treated with EV + P. Most patients presented within the first 2 cycles of EV + P with widespread morbilliform eruption on the trunk and extremities; more severe presentations tended to be desquamative and involve the intertriginous areas, head, neck, and mucosa. Histopathologic examination revealed spongiosis and perivascular inflammation in milder cases, while more severe cases demonstrated interface dermatitis, subepidermal splitting, and full-thickness epidermal necrosis as may be seen in SJS/TEN. Two Grade 4 presentations also had eccrine gland involvement, suggestive of a direct cytotoxic effect as seen in TEC. Although our data are limited, early biopsy in patients with rash may potentially help predict the severity of cutaneous toxicity, particularly if features such as eccrine involvement and lichenoid dermatitis are present. Further studies are warranted to explore histologic markers that may aid in identifying patients at risk for severe reactions.

Despite the severity of the cutaneous reactions, most patients demonstrated signs of recovery within 10 days of admission, indicating a positive response to systemic steroids (*n* = 6). These patients eventually achieved complete resolution of symptoms within 60 days. Among those with severe presentations (P_6-8_), 2 patients who received systemic steroids and either IVIG or rifampin fully recovered. However, P_8_, who received only IVIG, did not improve and expired 1 day after transfer to our institution, prior to initiation of systemic steroids. Notably, P_6,7_ achieved complete cancer remission without treatment rechallenge or subsequent chemotherapy, supporting emerging evidence that cAE severity may correlate with cancer treatment efficacy.^12^

Management of cutaneous toxicities in patients receiving EV + P is largely extrapolated from experience with traditional chemotherapy or ICI monotherapy. Initial treatment includes intense supportive care, specialist evaluation, symptomatic management with emollients and antihistamines, and medical therapy with topical and systemic steroids.^9^^,^^13^ Patients with severe desquamative reactions are best managed in an intensive care setting.^12^ Additional interventions should be tailored to the underlying morphology, including prompt initiation of higher dose steroids (≥1-2 mg/kg) for severe reactions. Notably, one patient (P_7_) with a SCORTEN = 6 was successfully treated with supportive care, systemic steroids and rifampin, with the intent of capitalizing on rifampin’s CYP 3A4 inducer properties to theoretically reduce exposure to cytotoxic MMAE via increased metabolism.^14^ Clinical assays for tissue and plasma MMAE concentrations are notably unavailable. We anticipate that diagnosis and individualized management of toxicity could improve with pharmacokinetic and informed clinical trials that explore drug–drug interactions for treating severe cAEs. Furthermore, adjusting EV dosage to match tumor burden may help reduce off-target effects.

Anticancer therapy should be withheld in patients with Grade 3 reactions until improvement to a Grade 1 reaction and ≤10 mg prednisone daily, at which time EV or P may be reintroduced.^9^ Permanent discontinuation of EV + P is indicated in Grade 4 reactions.^7^^,^^9^^,^^12^ EV + P treatment interruption was necessary in all cases and patients with milder presentations (*n* = 5, P_1-5_) resumed EV + P or P monotherapy after 5-29 days, supporting cautious rechallenge in appropriate patients. Subsequent flares were common in patients who resumed EV + P; ongoing and anticipatory management may be necessary. Notably, patients who were switched to ICI monotherapy did not experience subsequent cAEs. More research is needed to determine the effect of EV + P treatment interruption on cancer status and implications for chemotherapeutic rechallenge.

Efficacious and tolerable treatments for patients with la/mUC are needed. EV is a first-in-class nectin-4-directed antibody-drug conjugate that has shown progression-free and overall survival benefit over conventional chemotherapy, and EV + P combination therapy is now widely accepted as a first-line therapy in advanced urothelial cancers. Inpatient dermatologists can expect to see a proportionate increase in consultations for cAEs in EV + P treated patients. By understanding the pathophysiology of EV + P-related cAEs and having protocols for prevention and management, oncologists and dermatologists will be better equipped to educate, monitor and manage patients to minimize toxicity, maximize clinical benefit, and maintain quality of life. Further study is needed to identify optimal treatments and explore factors that may predispose patients to severe drug reactions.

## Conflicts of interest

Dr Hoimes has received Duke research funding and endorses consulting relationship with SEAGEN and MERCK. Drs Shearer, Sarah Myers, Whitley, and Authors Emma Myers, Liu, and Schneider have no conflicts of interest to declare.
